# Task-State Cortical Motor Network Characteristics by Functional Near-Infrared Spectroscopy in Subacute Stroke Show Hemispheric Dominance

**DOI:** 10.3389/fnagi.2022.932318

**Published:** 2022-06-24

**Authors:** Ziwen Yuan, Weiwei Xu, Jiameng Bao, Hui Gao, Wen Li, Yu Peng, Lisha Wang, Ye Zhao, Siming Song, Jin Qiao, Gang Wang

**Affiliations:** ^1^Key Laboratory of Biomedical Information Engineering of Ministry of Education, Institute of Biomedical Engineering, School of Life Sciences and Technology, Xi’an Jiaotong University, Xi’an, China; ^2^Department of Rehabilitation, First Affiliated Hospital of Xi’an Jiaotong University, Xi’an, China; ^3^College of Biomedical Engineering and Instrument Science, Zhejiang University, Hangzhou, China

**Keywords:** hemispheric dominance, fNIRS, brain network, motor, task-state, stroke

## Abstract

**Background:**

There was a reorganization of the brain network after stroke. Some studies have compared the characteristics of activation or functional connectivity (FC) of cortical and subcortical regions between the dominant and non-dominant hemisphere stroke.

**Objectives:**

To analyze hemispheric dominance differences in task-state motor network properties in subacute stroke by functional near-infrared spectroscopy (fNIRS).

**Materials and Methods:**

Patients with first ischemic stroke in the basal ganglia within 1–3 months after onset and age- and sex-matched right-handed healthy subjects (HS) were enrolled. fNIRS with 29 channels was used to detect the oxyhemoglobin concentration changes when performing the hand grasping task. Activation patterns of motor cortex and two macroscale and two mesoscale brain network indicators based on graph theory were compared between dominant and non-dominant hemisphere stroke.

**Results:**

We enrolled 17 subjects in each of left hemisphere stroke (LHS), right hemisphere stroke (RHS), and HS groups. Both patient groups showed bilateral activation. The average weighted clustering coefficient and global efficiency of patients were lower than those of healthy people, and the inter-density was higher than that of the HS group, but the significance was different between LHS and RHS groups. The intra-density changes in the RHS group were opposite to those in the LHS group. The correlation between mesoscale indicators and motor function differed between dominant and non-dominant hemisphere stroke.

**Conclusion:**

The changes in macroscale cortical network indicators were similar between the two patient groups, while those of the mesoscale indicators were different. The mesoscale brain network characteristics were affected by the severity of dysfunction to varying degrees in the LHS and RHS patients.

## Introduction

The brain has plasticity, and makes adaptive changes after stroke, resulting in the reorganization and compensation of neural networks (Carlson et al., 2020). The mechanism of motor function rehabilitation and brain motor network remodeling after stroke has always been a research hotspot. The activation of the primary motor cortex (M1) of the affected hemisphere was weakened when the affected hand performs simple movements after stroke, while the ipsilateral premotor cortex (PMC), supplementary motor area (SMA), and the contralesional motor areas were activated to varying degrees according to the location and size of the injury (Shimizu et al., 2002; Calautti et al., 2003). Its physiological basis may be that in addition to most of the projection fibers from the M1 area, the corticospinal tract also includes projections from the PMC, SMA, parietal lobes, and other cortices, and about 10–15% of the corticospinal tract fibers do not cross (Kato et al., 2002).

Based on the abovementioned physiological mechanisms, neuromodulation techniques such as repetitive transcranial magnetic stimulation (rTMS) have been widely used to promote brain network remodeling after stroke. At present, the most commonly used strategy is the excitatory rTMS on the ipsilesional motor cortex and/or the inhibitory rTMS on the contralesional hemisphere (Harvey et al., 2018; Watanabe et al., 2018; Yang et al., 2021). However, some patients have poor treatment effects. This may be related to the heterogeneity of stroke severity, course, lesion location, and stroke type, and stroke patients with different characteristics may respond differently to stimuli. For example, the state and plasticity of the brain are different during different phases (acute, subacute, or chronic) of stroke (Miyai et al., 2003; Wang et al., 2010; Park et al., 2011). In 2015, a systematic review including 37 studies on the effect of rTMS on the upper extremity function after stroke found that low-frequency rTMS on the uninjured hemisphere had a better effect on chronic stroke, and high-frequency rTMS on the injured hemisphere had a better effect on acute stroke (Ludemann-Podubecka et al., 2015a). One study compared the effect of rTMS in patients with different infarct sites and found that facilitative rTMS on the ipsilesional M1 was more effective in patients with subcortical infarction than in patients with cortical infarction (Ameli et al., 2009). In addition, the severity of the disease is a key factor affecting the efficacy. In severely injured patients, inhibition of the contralesional motor area may not be beneficial (McCambridge et al., 2018).

In recent years, studies have found that whether the lesion is located in the dominant hemisphere also affects the effect of rTMS treatment. Ludemann-Podubecka et al. (2015b) found that low-frequency rTMS on the contralesional cortex only improved hand function in patients with lesions in the dominant hemisphere. Therefore, it is necessary to analyze the characteristics of the brain network in the dominant or non-dominant hemisphere stroke to guide rehabilitation treatment. At present, studies have used functional magnetic resonance imaging (fMRI), functional near-infrared spectroscopy (fNIRS), and electroencephalography (EEG) to compare the characteristics of activation or functional connectivity (FC) of cortical and subcortical regions between the dominant and non-dominant hemisphere stroke. For example, two fMRI-based studies analyzed cortical and subcortical activation signatures in acute and chronic stroke patients and found that they were dependent on hemisphere (Liew et al., 2018; Vidal et al., 2018). Caliandro et al. (2017) analyzed the small-world properties of resting-state brain networks by EEG in acute stroke patients, and found similar outcomes for left and right hemisphere strokes (RHSs) in delta and alpha rhythms, but in the theta rhythm, bilaterally decreased small-worldness was observed only in the left hemisphere stroke (LHS). Another two studies used fNIRS to investigate post-stroke FC changes and also found that there were differences in whether or not the dominant hemisphere stroke (Lu et al., 2019; Arun et al., 2020). However, there was no study to compare the task-state brain network characteristics between left and RHS. Considering the needs of the motor task and the advantages of fNIRS (portability, tolerance to head motion, better spatial resolution than EEG, and better temporal resolution than fMRI) (Harrivel et al., 2013; Chen et al., 2020), we used fNIRS to capture hemispheric dominance differences in task-state motor network characteristics in patients with subacute ischemic stroke.

## Materials and Methods

### Participants

While brain functional compensation in the acute phase of stroke is passive or transient (Bonkhoff et al., 2020), we included stroke patients in the subacute phase to observe the actively established and relatively stable functional connections. In addition, because hemodynamic responses measured by fNIRS may be affected by brain artery occlusion and lesion location, only patients with stroke in the basal ganglia were enrolled. The main inclusion criteria were as follows: (1) Age 40–79 years; (2) First ischemic stroke in the basal ganglia within 1–3 months after onset; (3) Right-handed according to the Edinburg Handedness scale; (4) Brunnstrom stage (hand) II or above; and (5) Can cooperate with the fNIRS assessment. Exclusion criteria were as follows: (1) Be afraid of the dark; (2) Severely impaired cognition or inability to pay attention to the computer screen; and (3) Visible movements of other limbs other than the task hand were observed during the task state collection. A total of 28 LHS and 31 RHS patients were screened from our ongoing trial “Dynamic Individualized rTMS Based on fNIRS” in our rehabilitation center (*clinicaltrials.gov*: NCT04617366), and they were divided into LHS group and RHS group. In addition, age- (difference <5 years) and sex-matched right-handed healthy subjects (HS) without motor dysfunction were recruited.

The study was approved by the Ethics Committee of the First Affiliated Hospital of Xi’an Jiaotong University on 25 March 2020 (No. 2020 G-103), and all patients or their authorized agents have signed informed consent.

### Functional Near-Infrared Spectroscopy Measurement

**FIGURE 1:**
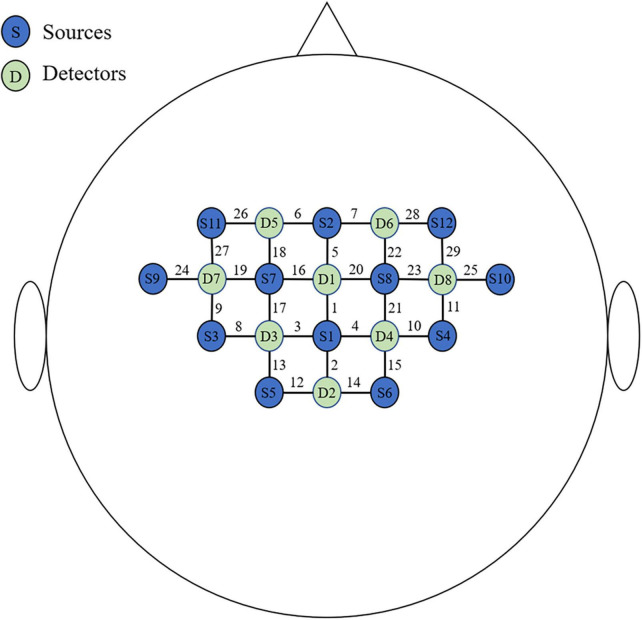
The montage of the probes.

The acquisition was performed using the NirSmart system (Danyang Huichuang Medical Equipment Co., Ltd., China) having 12 sources and eight detectors at a sampling rate of 10 Hz in a quiet and relatively darker room. The wavelengths used were 730 and 850 nm. The montage of the probes is shown in Figure 1. The S1 probe was placed at the Cz point (10/20 international system), and a total of 29 channels were formed with a fixed 3 cm inter-probe distance. The area covered by the probes included three regions of interest (ROI) both in the left and right hemispheres: (SMA; Left: Channel 1, 5, 6, 16; Right: Channel 1, 5, 7, 20), premotor area (PMC; Left: Channel 17, 18, 19, 24, 26, 27; Right: Channel 21, 22, 23, 25, 28, 29), and primary sensorimotor area (SM1; Left: Channel 2, 3, 8, 9, 12, 13; Right: Channel 2, 4, 10, 11, 14, 15). The Fugl-Meyer Assessment (FMA) of upper limbs (FMA-UL) was performed on the day of the fNIRS measurement. A dedicated physician was assigned to perform the FMA assessment for all patients.

### Task Paradigm

All subjects were asked to remain at a resting state before performing the measurement. After communicating with the subjects about the measurement procedure, speaking was prohibited. In the resting state, the subjects remained relaxed in a sitting position, with their palms up on the legs, and the photograph of the stationary hand was observed on the computer screen for 180 s. Next was the task state, in which the patient only performed the affected hand grasping, and the HS group performed both the left (HS-L) and right hand (HS-R) tasks. The task included five trails with no gaps, and each trial included a 25 s resting block and a 20 s task block. The rules of resting-block were the same as the resting-state, and in the task-block, the subjects were taught to follow the video to perform the hand grasping task (1 Hz) while keeping the ipsilateral upper and forearm and other limbs relaxed and still.

### Data Pre-processing

The NirSpark software package (Danyang Huichuang Medical Equipment Co., Ltd., China) was used to process fNIRS data. First, we converted the raw optical intensity data to optical density data. Second, we used a bandpass filter at 0.01–0.20 Hz to remove physiological noise (heart rate and respiration). Then, spline interpolation was used three times to remove motion artifacts (Scholkmann et al., 2010). Finally, the denoised optical density data were converted into hemoglobin concentration data. It has been shown that oxyhemoglobin (HbO) concentration was considered to have a better signal-to-noise ratio than deoxyhemoglobin (HbR) concentration (Strangman et al., 2002); therefore, we used HbO concentration for subsequent analysis.

### Activation and Lateralization Index

The general linear model (GLM) was used to analyze hemodynamic time series in task blocks to evaluate the activations. The significantly activated channels in the task state compared with the resting state were obtained by one-sample *t*-test and FDR correction.

The lateralization index (LI) was calculated based on the mean HbO concentration in the MS1 region of both hemispheres. For example, the LI formula during the left hand task is given as follows:


(1) $$\text{LI}=\frac{\mathrm{Right}-\mathrm{Left}}{\text{Right}+\text{Left}}$$

The range of LI scores is −1 to 1, where 1 means only right hemisphere activation, and −1 means only left hemisphere activation. Besides, the activation patterns were categorized as bilateral (∣ LI ∣ ≤ 0.1), hemisphere dominant (0.1 < ∣ LI ∣ < 0.2), or hemisphere lateralized (∣ LI ∣ ≥ 0.2) (Jansen et al., 2006).

### Brain Network Analysis

We conducted brain network analysis by graph theory, treating the 29 channels as nodes in a graph. First, Pearson correlation analysis was used to calculate the correlation coefficient of time series in task blocks between 29 channels (Equation 2) (Yu et al., 2020). Then, a 29 × 29 correlation matrix (or FC matrix) was established with the averaged correlation coefficients of the five task blocks. A Fisher’s z transformation was further applied to the correlation matrix to convert the sampling distribution of ρ to the normal distribution (Nguyen et al., 2018).


(2) $$\rho_{i,j}=\frac{\sum_{n=1}^{N}\left(y_{i,n}-\bar{y_{ı}}\right)\,\left(y_{j,n}-\bar{y_{ȷ}}\right)}{\sqrt{\sum_{n=1}^{N}{\left(y_{i,n}-\bar{y_{ı}}\right)}^{2}}\,\sqrt{\sum_{n=1}^{N}{\left(y_{j,n}-\bar{y_{ȷ}}\right)}^{2}}}$$

where *y*_*i*_ represents the time series of all sampling points (*N*) of the channel *i*, *y*_*i,n*_ represents the time series of a sampling point *n* of the channel *i*; *ρ_*i,j*_* represents the Pearson correlation coefficient between the channel *i* and *j*, the range is −1 to 1, where 1 means that the two channels have a perfectly positive correlation and −1 means that the two channels have a completely negative correlation.

Next, the proportional thresholding (10–50% network sparsity) was applied to exclude weak or irrelevant FC from the analysis of the graph (Duan et al., 2012), and a total of nine brain networks under corresponding sparsity thresholds were constructed. When the percentile of the correlation coefficient between two nodes among all correlation coefficients in the network was greater than the threshold, the connections between nodes were considered valid. Each remaining connection was identified as an edge of the graph, and the correlation coefficient was the weight of the connection between nodes (*w*_*i,j*_); thus, an undirected weighted graph was constructed. Four most commonly used graph-theoretic indicators were selected to describe the network characteristics: macroscale average weighted clustering coefficient (C) and global efficiency (E) (Mazrooyisebdani et al., 2018) and the inter-density (K-inter) and intra-density (K-intra) at the mesoscale (De Vico Fallani et al., 2013).

**(1) Average weighted clustering coefficient (C)**

The C represents the degree to which the nodes in the graph tend to cluster and is a measure of the local separation of the graph. The higher is the C, the higher is the degree of segregation (or specialization). The formula of weighted clustering coefficient of a node *i* is given as follows:


(3) $$c_{i}=\frac{1}{k_{i}\,\left(k_{i}-1\right)}\,\sum_{j,k}{\left(w_{i,j}\,w_{j,k}\,w_{k,i}\right)}^{\frac{1}{3}}$$

where *k*_*i*_ represents the degree of a node *i*.

Then, the C of a network is calculated as follows:


(4) $$C=\frac{1}{N}\,\sum_{i\in N}c_{i}$$

**(2) Global efficiency (E)**

The E is the inverse property of the shortest path length, which represents the ability of nodes to transmit information and is a measure of the level of integration of the network. The higher is the E, the stronger is the ability of the network to transmit information. The E of a network is calculated as follows:


(5) E=1N⁢(N-1)⁢∑i∈N∑j∈N,j≠idi⁢j-1

where *d*_*ij*_ is the shortest path length from node *i* to *j* (Equation 6). The higher is the correlation coefficient between two nodes, the shorter is the path length, which is converted by reciprocal mapping of the weights of connections (*w*_*i,j*_).


(6) di,j=min⁢{wi⁢j-1}

**(3) Inter-density (K-inter)**

The K-inter is defined as the ratio of the actual number of connections among all possible connections between two sets (Equation 7). By definition, the K-inter ranges from 0 to 1, with higher K-inter reflecting more inter-hemispheric connectivity.


(7) $$\begin{array}{c} K-i\,n\,t\,e\,r=\frac{1}{N_{S}^{2}}\,\sum_{i,j\in S_{A\,h\,e\,m\,i,U\,h\,e\,m\,i}}a_{i,j} \end{array}$$

where *S* (or *S_*Ahemi*_,S_*Uhemi*_)* represents the set of nodes within the affected hemisphere (Ahemi) and unaffected hemisphere (Uhemi), *N*_*S*_ represents the total number of nodes in the two sets [*N(S_*Ahemi*_)* = *N(S_*Uhemi*_)* = *N*_*S*_ = 13], and *a*_*i,j*_ represents the connection state of the nodes *i* and *j*. The rule is given as follows:


(8) $$a_{i,j}=\left\{ \begin{array}{cc} 0, & w_{i,j}=0\\ 1, & e\,l\,s\,e \end{array}\right.$$

**(4) Intra-density (K-intra)**

The K-intra is defined as the proportion of actual connections among all possible connections within a set [*N(S_*Ahemi*_)* = *N(S_*Uhemi*_)* = *N*_*S*_ = 16], which also ranges from 0 to 1 (Equation 9). Higher K-intra reflects more intra-hemispheric connectivity.


(9) $$\begin{array}{c} K-i\,n\,t\,r\,a\,\left(S\right)=\frac{2}{N_{S}\,\left(N_{S}-1\right)}\,\sum_{i\neq j\in S}a_{i,j} \end{array}$$

**(5) Area under the curve**

For each brain network indicator, a curve was plotted with the ordinate as the network indicator value and the abscissa as the 10–50% sparsity stepped by 5%. Then, we calculated the area under curves (AUCs) over the whole sparsity range for the above network indicators, which may represent the average levels under different sparsity and was used for the following analysis.

### Statistical Analysis

The comparison of measurement data between the two groups was performed by two independent-samples *t*-test or Kruskal–Wallis rank-sum test; the comparison of count data was performed by chi-square test or Fisher’s exact test. After testing the normal distribution or homogeneity of variance, the comparison of the AUCs for the brain network indicators between patients and HS was performed using two independent-samples *t*-test. Then, Spearman correlation analysis (*r*) was used to analyze the correlation between the AUCs and FMA-UL (and FMA-hand). Furthermore, the generalized additive model (GAM) was used to fit the AUCs and FMA-UL (or FMA-hand) to a smooth curve to evaluate whether there is a threshold effect. Two-sided α = 0.05 was used. The sample size was set to be larger than 15 in each group according to the previous related studies. We used Empower (R) (X&Y solutions, Inc., Boston, MA, United States),^1^ R software, version 3.1.2,^2^ and MATLAB version 2019b (MathWorks, Inc., Natick, MA, United States) for the statistical analyses.

## Results

### Participants’ Characteristics

We enrolled 17 right-handed patients with subcortical stroke both in the LHS and RHS groups and 17 HS from November 2020 to July 2021. The mean age of patients was 60.7 ± 9.6 years in both groups, and 56.1 ± 4.3 years in the HS group (*p* = 0.022). Although there was statistical difference of age between patients and HS, it was recognized that the difference of less than 5 years did not have clinical significance. There were five female patients in each group (29.4%). Most patients presented with moderate motor dysfunction in the upper limb. The mean FMA-UL score was 35.8 ± 22.2 (hand part: 7.5 ± 4.6) in the LHS group, and 35.2 ± 17.1 (hand part: 6.2 ± 4.2) in the RHS group, respectively. No significant difference was observed in time from onset to admission, the history of hypertension and diabetes, and other baseline characteristics between the two stroke groups (Table 1).

**TABLE 1:** Baseline characteristics of stroke patients^a^.

	LHS	RHS	*p*
*N*	17	17	
Age, years	60.2 (8.3)	61.2 (11.0)	0.767^b^
Female	5 (29.4%)	5 (29.4%)	1.000^c^
Time of onset, days	38 (35–47)	34 (32–45)	0.369^d^
FMA-UL	34 (17–62)	33 (21–47)	0.959^d^
FMA-hand	6 (3–13)	4 (3–9)	0.367^d^
Hypertension	12 (70.6%)	13 (76.5%)	1.000^c^
Diabetes	3 (17.6%)	2 (11.8%)	1.000^c^

*LHS, left hemisphere stroke; RHS, right hemisphere stroke; FMA, Fugl-Meyer Assessment; FMA-UL, Fugl-Meyer Assessment of upper limbs.*

*^a^Values are presented as mean (SD), median (Q1–Q3), or N (%).*

*^b^Evaluated using t-test.*

*^c^Evaluated using Fisher’s exact test.*

*^d^Evaluated using Kruskal-Wallis rank sum test.*

### Activation and Lateralization Index

**FIGURE 2:**
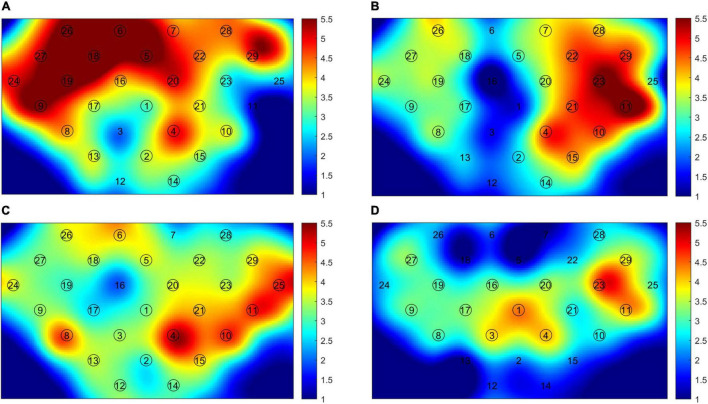
The cerebral cortex activation map. **(A)** Right-hand task of healthy subjects (HS); **(B)** left-hand task of HS; **(C)** right-hand task of left hemisphere stroke (LHS) patients; **(D)** left-hand task of right hemisphere stroke (RHS) patients. The circled channels are significantly activated channels.

In the HS group, the activation of the contralateral hemisphere was dominant during the hand grasping task. The mean LI of SM1 was 0.436 (SD: 0.240) for the left-hand task, and 0.138 (SD: 0.002) for the right-hand task. Both patient groups showed activation of bilateral hemispheres (LI of SM1: RHS, −0.056 ± 0.002; LHS, 0.015 ± 0.111). We may see that absolute values of LI were both less than 0.1, so the activation pattern was thought to be bilateral (Jansen et al., 2006). In the LHS (dominant side) group, the contralesional PMC and bilateral SM1 were mainly activated in the right-hand grasping task, while the ipsilesional PMC and bilateral SMA, SM1 were mainly activated in the RHS (non-dominant side) group. The significantly activated channels are shown in Figure 2.

### Brain Network Analysis

**FIGURE 3:**
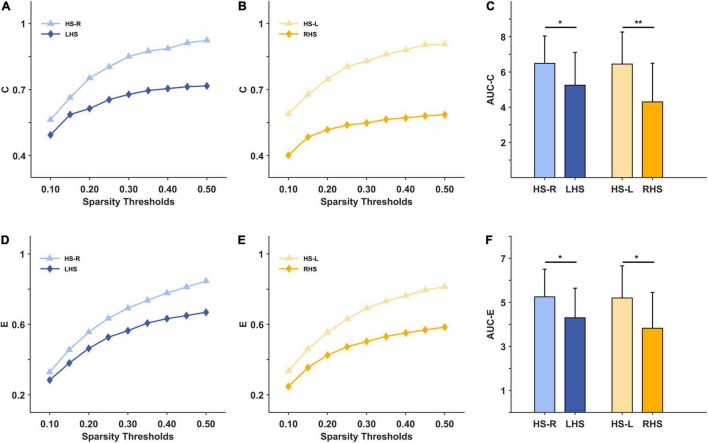
The macroscale brain network indicators under 10–50% sparsity and their area under curves (AUCs) for patients and healthy subjects (HS). **(A)** the average weighted clustering coefficient under 10%-50% sparsity for HS group (right hand task) and LHS group; **(B)** the average weighted clustering coefficient under 10%-50% sparsity for HS group (left hand task) and RHS group; **(C)** the AUCs over the whole sparsity range for the average weighted clustering coefficient in the HS, LHS and RHS groups. **(D–F)** are for the indicator global efficiency. *p < 0.05, **p < 0.01.

**FIGURE 4:**
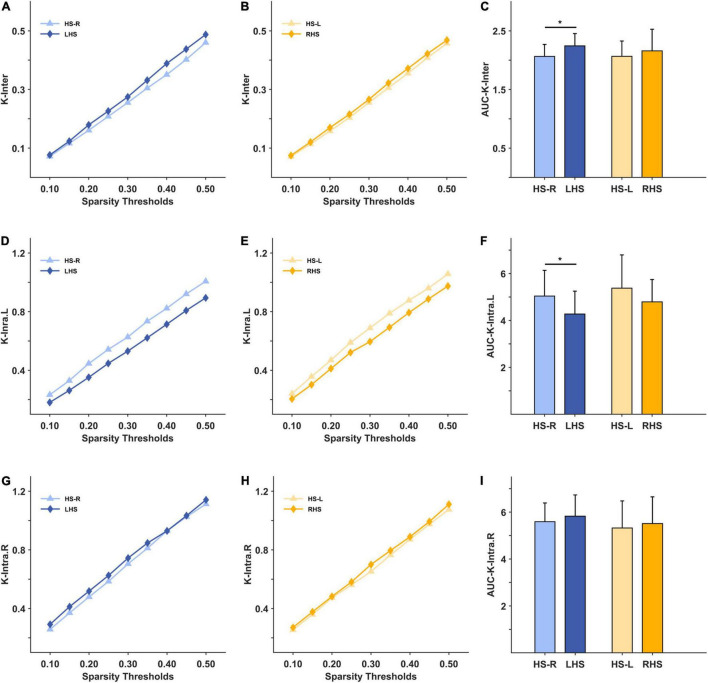
The mesoscale brain network indicators under 10–50% sparsity and their area under curves (AUCs) for patients and healthy subjects (HS). **(A)** The inter-density under 10%–50% sparsity for HS group (right hand task) and LHS group; **(B)** the inter-density under 10%–50% sparsity for HS group (left hand task) and RHS group; **(C)** the AUCs over the whole sparsity range for the inter-density in the HS, LHS and RHS groups. **(D–F)** are for the indicator intra-density of left hemisphere; **(G–I)** are for the indicator intra-density of right hemisphere. *p < 0.05.

Under the 10–50% sparsity threshold, the average weighted clustering coefficient and global efficiency of the two groups of patients were both lower than those of healthy people (Figures 3A,B,D,E). The difference was significant for the LHS group (C: *p* = 0.044, E: *p* = 0.040), and for the RHS group (C: *p* = 0.004, E: *p* = 0.014) (Figures 3C,F). The inter-density in the two groups of patients was higher than that of the HS group, and the difference was significant only in the LHS group (LHS: *p* = 0.016, RHS: *p* = 0.396) (Figures 4A–C). The intra-density in the left hemisphere (K-intra.L) of the LHS group was lower than that of the HS group (*p* = 0.038, Figures 4D,F); the intra-density in the right hemisphere (K-intra.R) was higher than that of HS group (*p* = 0.427 Figures 4G,I). However, the intra-density changes in the RHS group were opposite to those in the LHS group. That is, the intra-density in the affected hemisphere was higher than that of the HS group (*p* = 0.639, Figures 4H,I), while that in the unaffected hemisphere was lower (*p* = 0.168, Figures 4E,F).

### Correlation With Fugl-Meyer Assessment

Correlation analysis was made between AUCs of the network indicators and FMA-UL or FMA-hand (Table 2). At large scales, the higher C and E values reflected greater motor function of the upper limb in both groups. But only indicator E in the LHS group was significantly correlated with FMA-UL (*r* = 0.524, *p* = 0.031). At the mesoscale, inter-density and FMA-UL were positively correlated in the LHS group (*r* = 0.304, *p* = 0.235) and slightly correlated in the RHS group (*r* = −0.027, *p* = 0.918). In the LHS group, there was a negative correlation between the K-inter.R and FMA-UL (*r* = −0.262, *p* = 0.311). The correlation between other intra-density and FMA-UL was small. Correlation results between brain network indicators and FMA-hand were similar. Among them, the intra-density of the contralesional hemisphere was negatively correlated with the FMA-hand in the RHS group (*r* = −0.345, *p* = 0.175). It can be seen from the above results that the correlation between each network indicator and FMA in the LHS group was higher than that in the RHS group. Was that because there was a non-linear correlation?

**TABLE 2:** Spearman correlation coefficients (*r*) of the AUC of brain network indicators and FMA.

		FMA-UL		FMA-hand	
		*r*	*p*	*r*	*p*
C	LHS	0.367	0.147	0.324	0.205
	RHS	0.253	0.3288	0.319	0.212
E	LHS	0.524	0.031	0.458	0.064
	RHS	0.326	0.201	0.408	0.104
K-inter	LHS	0.304	0.235	0.266	0.303
	RHS	−0.027	0.918	0.100	0.702
K-intra.L	LHS	0.098	0.708	0.036	0.891
	RHS	−0.115	0.659	−0.345	0.175
K-intra.R	LHS	−0.262	0.311	−0.210	0.419
	RHS	0.157	0.547	0.142	0.586

*AUC, area under curve; LHS, left hemisphere stroke; RHS, right hemisphere stroke; FMA, Fugl-Meyer Assessment; FMA-UL, Fugl-Meyer Assessment of upper limbs; C, average weighted clustering coefficient; E, global efficiency; K-inter, inter-density; K-intra.L, intra-density of left hemisphere; K-intra.R, intra-density of right hemisphere.*

**FIGURE 5:**
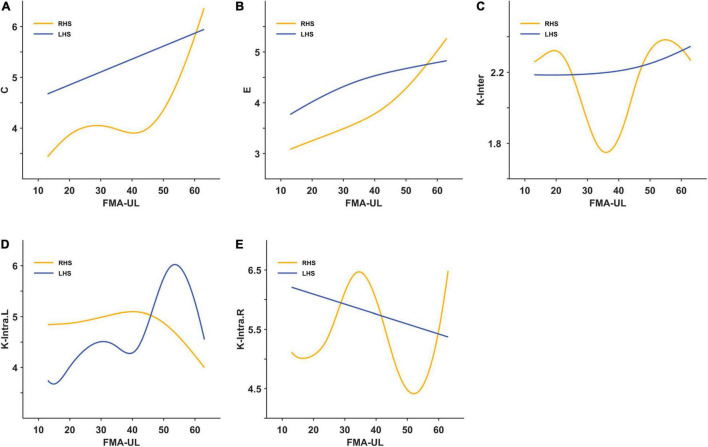
The smooth curve fitting between area under curves (AUCs) of the brain network indicators and Fugl-Meyer Assessment of upper limbs (FMA-UL). **(A)** For the average weighted clustering coefficient; **(B)** for the global efficiency; **(C)** for the inter-density; **(D)** for the intra-density of left hemisphere; **(E)** for the intra-density of right hemisphere.

Through smooth curve fitting, the relationship between average weighted clustering coefficient or global efficiency and FMA-UL of the two groups has the same trend as the above correlation analysis (Figures 5A,B). Most of the mesoscale network parameters in the LHS group remained approximately linear with the FMA-UL, except for the K-intra.L. When motor function was poor (FMA-UL < 52), a better function was associated with larger intra-density of the left hemisphere (*r* = −0.196, *p* = 0.541). But when FMA-UL ≥ 52, the relationship was the opposite (*r* = −0.618, *p* = 0.191) (Figure 5D). That is, only the intra-density of the left hemisphere was affected by function severity in the LHS group. However, all of the relationships between inter-density or intra-density and FMA-UL had threshold effects in the RHS group (Figures 5C–E). When the FMA-UL was less than 33, there was a negative correlation between inter-density and FMA-UL (*r* = −0.303, *p* = 0.429), but when FMA-UL ≥ 33, higher inter-density values reflected greater motor function of upper limb (*r* = 0.571, *p* = 0.084). The relationship between K-intra.L and FMA-UL was also affected by the severity of motor dysfunction. When motor function was poor (FMA-UL < 42), the value of K-intra.L decreases with poorer motor function was better (FMA-UL ≥ 42), the value of K-intra.L increased (*r* = −0.714, *p* = 0.088). When 33 ≤ FMA-UL < 49, there was a significant negative correlation between ipsilesional intra-density and upper limb motor function (r = −0.883, *P* = 0.009), while there was a positive correlation when FMA-UL < 33 (*r* = 0.555, *p* = 0.121) and ≥ 49 (*r* = 0.800, *p* = 0.333).

## Discussion

Hemiplegia after stroke is especially slow in hand motor function recovery, so we tried to guide the post-stroke hand function rehabilitation by analyzing the motor-related network characteristics during the hand grasping task. Our study found that both left and RHS exhibited bilateral activation during the affected hand task, but the activation regions were different. Miyai et al. (2003) used fNIRS to dynamically observe eight stroke patients with an average onset of 3 months for 2 months and found that the activation of contralesional SM1 gradually decreased, while the activation of ipsilesional PMC gradually increased. Wang et al. (2010) observed the complete motor network topological changes from the acute phase to the chronic phase after stroke using resting-state fMRI. The connections between the ipsilesional M1 and contralesional primary sensory area (S1), ventral PMC (PMv), dorsal PMC (PMd), and M1, between the ipsilesional PMv and the contralesional dentate nucleus of the cerebellum, and between the ipsilesional PMd and the contralesional superior parietal lobule (SPL) were significantly enhanced. These studies confirmed the role of SM1 and PMC activation in neurological compensation after stroke. In our study, we also found that both groups of stroke patients had compensatory activation of contralesional SM1, but the LHS was dominated by the contralesional PMC activation, and the RHS was dominated by the ipsilesional PMC activation.

Several published studies comparing hemispheric dominance have also demonstrated whether or not the dominant hemisphere stroke differs in brain network remodeling after stroke. Vidal et al. (2018) used fMRI to analyze the motor network activation during shoulder forward flexion-maintenance-release for 10 s each in the acute phase of stroke. The brain activation for RHS occurred in bilateral motor cortex, especially in SMA; while the LHS showed the ipsilesional cortical activation during forward flexion, and bilateral activation when put down (Vidal et al., 2018). Liew et al. (2018) analyzed brain activation during motor observation by fMRI in chronic-phase stroke patients. The RHS patients showed activation of left supramarginal gyrus and bilateral M1, SPL, and occipital lobe when observing left-hand movements, while the activation of left PMC and bilateral M1, supramarginal gyrus, and occipital lobes was noted in the LHS patients during right-hand movement (Liew et al., 2018). Although the brain activation also correlated with task paradigms (Allison et al., 2000; Nirkko et al., 2001; Grefkes et al., 2008), the activation characteristics of LHS and RHS were different under the same motor task.

At the macroscale level, our study found that the clustering coefficient and global efficiency of the patients were lower than those of the HS group, but the significance at each threshold was different. Caliandro et al. (2017) analyzed the small-world characteristics of cortical connectivity in acute stroke and came to a similar conclusion: the segregation and integration abilities of both dominant and non-dominant hemisphere stroke patients were lower than healthy people in all of the δ/ θ/ α_2_ rhythms, but it varied in significance in different rhythms. At mesoscale, the inter-density of the patients was higher than that of HS in our study. The intra-density of the affected hemisphere was decreased and that of the unaffected hemisphere was increased in the LHS group, yet the results of the RHS group were opposite. We may infer that the motor function of the dominant hemisphere stroke was mainly compensated by the motor network of the contralesional hemisphere, while the non-dominant hemisphere stroke was compensated by the bilateral motor networks. Arun et al. (2020) compared the resting-state FC of left and RHS patients at 4–8 weeks using fNIRS; the findings of this group were similar to ours: there was decreased FC in the left hemisphere and increased FC in the right hemisphere for LHS (dominant side), and increased intrahemispheric (both sides) and interhemispheric FC for RHS.

It was considered that activation in the ipsilesional hemisphere predicted better motor recovery (Favre et al., 2014), whereas hyperactivation of the contralesional hemisphere predicted persistent neurological and motor deficits (Rehme and Grefkes, 2013; Grefkes and Fink, 2014). However, we found that brain network parameters in RHS (non-dominant side) were affected by the severity of dysfunction. When the dysfunction was higher (FMA-UL < 33), the worse motor function needed greater interhemispheric connections, but less intra-density of both hemispheres. It showed that the worse is the function, the more information exchange was needed from the contralesional hemisphere to the ipsilesional side, but the information exchange was less dependent between the three motor areas in the two hemispheres, so the compensation of the contralesional hemisphere was more critical when the motor function was poor. Whereas the brain network results were reversed when the dysfunction was mild (33 ≤ FMA-UL < 49), and smaller FMA-UL values reflected smaller inter-density and larger intra-density of the affected hemisphere. Then, the compensation in the ipsilesional hemisphere seemed to be more important. Especially when FMA-UL ≥ 49, the information exchange between the motor areas in the ipsilesional hemisphere increased with the further improvement of motor function, also illustrating the importance of the compensation on the lesion side for the final functional recovery. Therefore, based on the above results, we inferred that the RHS patients with more severe dysfunction may not benefit from inhibiting the unaffected hemisphere, while those with milder dysfunction may benefit more by exciting the ipsilesional hemisphere. Yet for the LHS patients, larger intra-density of the affected hemisphere reflected better function when the impairment was severe (FMA-UL < 52). This phenomenon explained the importance of information exchange between the motor areas in the ipsilesional hemisphere, so these patients may benefit more by exciting the ipsilesional hemisphere. But when the left hemisphere was mildly injured (FMA-UL ≥ 52), there was less need for compensation from other motor areas. As demonstrated in the study by Sankarasubramanian et al. (2017), for patients with severe injury [fraction anisotropy (FA) of DTI > 0.5, FMA of the upper extremity (max = 36) < 26–28], facilitative rTMS on the contralesional PMd yielded more benefit than inhibitory rTMS on the contralesional M1, whereas it was the opposite for mild patients. McCambridge et al. (2018) also indicated that inhibition of the motor area of the unaffected hemisphere may not be beneficial in severely injured patients. What is more, Ludemann-Podubecka et al. (2015b) found that inhibitory rTMS on the contralesional hemisphere could only improve hand function in patients with dominant hemisphere stroke, and had no significant effect on non-dominant hemisphere stroke. Therefore, it is necessary to individualize neuromodulation according to the severity of dysfunction and the location of the lesion.

There were a few limitations of this study. The first was issues about the signal pre-processing of fNIRS. We set strict collection environment and conditions: a darker and quiet room, rest for 3 min, and restricting the movement of other limbs; then, the commonly used spline interpolation method was introduced to remove motion and blinking artifacts in fNIRS. However, the effect of removing some small artifacts was relatively poor. Besides, our probe coverage was limited to the common motor networks and failed to analyze the participation of other regions. Another limitation was that the sample size was small, and some results were not statistically significant, even with large effect sizes. On the one hand, we referred to the sample size of previous related studies; on the other hand, we strictly follow the inclusion and exclusion criteria to screen suitable patients to ensure high-quality fNIRS data. This study found differences in brain network characteristics between dominant and non-dominant hemisphere strokes. Yet, whether we can benefit from individualized treatment based on these results still needs to be verified by interventional studies.

## Conclusion

This is the first fNIRS-based study to compare the characteristics of macroscale and mesoscale brain networks between the dominant and non-dominant hemisphere stroke. Both LHS and RHS patients showed activation of bilateral hemispheres, but the activation regions were different. The average weighted clustering coefficient and global efficiency of both patient groups were lower than those of healthy people. The motor function execution of the LHS was mainly compensated by the motor network of the contralesional hemisphere, while that of the RHS was compensated by bilateral motor networks. Moreover, the mesoscale brain network characteristics were affected by the severity of dysfunction to varying degrees in the LHS and RHS patients. Our findings may help to develop an individualized neuromodulation strategy based on the patient’s stroke site and severity.

## Data Availability Statement

The original contributions presented in this study are included in the article/supplementary material, further inquiries can be directed to the corresponding authors.

## Ethics Statement

The studies involving human participants were reviewed and approved by the Ethics Committee of the First Affiliated Hospital of Xi’an Jiaotong University. The patients/participants provided their written informed consent to participate in this study.

## Author Contributions

ZY and GW conceived and designed the study. ZY, YP, LW, YZ, and SS screened participants. ZY, WX, JB, HG, and WL collected participants’ data and performed the statistical analysis. ZY wrote the first draft of the manuscript. JQ and GW reviewed and edited the manuscript. All authors contributed to the manuscript revision and read and approved the submitted version.

## Conflict of Interest

The authors declare that the research was conducted in the absence of any commercial or financial relationships that could be construed as a potential conflict of interest.

## Publisher’s Note

All claims expressed in this article are solely those of the authors and do not necessarily represent those of their affiliated organizations, or those of the publisher, the editors and the reviewers. Any product that may be evaluated in this article, or claim that may be made by its manufacturer, is not guaranteed or endorsed by the publisher.

## Footnotes

1
www.empowerstats.com

2
http://www.r-project.org
